# Comparative efficacy of surgery and radiotherapy in achieving local control in Kimura disease: a retrospective analysis of treatment outcomes

**DOI:** 10.3389/fonc.2025.1587648

**Published:** 2025-04-29

**Authors:** Wenlong Lv, Yanbin Chen, Xuezhen Wang, Shan Li, Feng Liu, Jinsheng Hong

**Affiliations:** ^1^ Department of Radiotherapy, Cancer Center, The First Affiliated Hospital of Fujian Medical University, Fuzhou, China; ^2^ Department of Radiotherapy, National Regional Medical Center, Binhai Campus of the First Affiliated Hospital, Fujian Medical University, Fuzhou, China; ^3^ Key Laboratory of Radiation Biology of Fujian Higher Education Institutions, The First Affiliated Hospital, Fujian Medical University, Fuzhou, China

**Keywords:** Kimura disease, surgery, radiotherapy, local control, recurrence

## Abstract

**Objective:**

To evaluate and compare the impact of surgery versus radiotherapy on local control rates in Kimura disease (KD).

**Methods:**

A retrospective analysis was conducted on 26 patients diagnosed with KD at the First Affiliated Hospital of Fujian Medical University from January 2001 to January 2024. Patients were categorized into four treatment groups: Primary Surgery (PS), Primary Radiotherapy (PR), Salvage Surgery (SS), and Salvage Radiotherapy (SR) following recurrence. Data on demographics, tumor characteristics, eosinophil counts (EO), disease duration, and radiation dose were collected. Univariate and multivariate analyses were performed to identify factors influencing local control rates.

**Results:**

The cohort had a mean age of 42.2 ± 17.7 years, with 24 male patients. Kaplan-Meier analysis revealed that radiotherapy provided superior local control compared to surgery, with significant differences between PS and PR (p = 0.047) and SS and SR (p < 0.001). No significant difference was found between PR and SR (p = 0.816). Multivariate analysis identified treatment modality as the strongest predictor of recurrence (HR: 0.062). Additionally, factors such as radiotherapy, bilateral involvement, and longer disease duration were associated with improved local control. Among radiotherapy patients, age, tumor number, tumor size, pre-treatment eosinophil count, radiotherapy dose, and disease duration significantly influenced prognosis.

**Conclusion:**

Radiotherapy is more effective than surgery in achieving local control of Kimura disease. Higher radiation doses may negatively impact outcomes, suggesting that a tailored, moderate-dose approach is optimal. Radiotherapy should be prioritized, particularly for recurrent or multifocal cases, offering a more reliable long-term treatment strategy than surgery.

## 1 Introduction

Kimura disease (KD) is a rare, chronic inflammatory disorder of unknown etiology, predominantly affecting young adult males of Asian descent (1, 2). Characterized by painless subcutaneous masses, particularly in the head and neck region involving the salivary glands and lymph nodes (3), KD is often accompanied by regional lymphadenopathy, peripheral eosinophilia, and elevated serum immunoglobulin E (IgE) levels (4). The male-to-female ratio typically ranges from 2:1 to 4:1, with a peak incidence observed between the ages of 20 and 40 (5, 6). While the precise incidence rate of KD remains unclear, regional studies suggest a strong predilection for Asian populations and a significant male predominance (5, 7). Epidemiological data regarding KD in non-Asian populations and female patients are currently limited, highlighting the need for further investigation.

Despite being a benign and often self-limiting condition, KD’s recurrent and chronic nature can cause ongoing discomfort and distress for patients (8). Treatment remains challenging, with traditional approaches including surgery, drug therapy (such as corticosteroids and immunosuppressants), and radiotherapy (9). Surgery, though standard, is associated with recurrence rates ranging from 25% to 100% (10). Recently, radiotherapy has gained attention as a promising alternative, with small case series and retrospective studies reporting excellent local control and minimal toxicity (11). However, the optimal long-term treatment strategy remains uncertain (12).

This report updates the experience of the First Affiliated Hospital of Fujian Medical University in managing KD (13), presenting further evidence supporting radiotherapy as a primary treatment option, either at initial presentation or following surgical recurrence.

## 2 Materials and methods

A retrospective analysis was performed on patients diagnosed with Kimura disease at the First Affiliated Hospital of Fujian Medical University between January 2006 and December 2022. All eligible patients had a confirmed pathological diagnosis. Patients without information on first-line treatment, unconfirmed diagnoses, or incomplete data were excluded. The demographic and clinical characteristics, including gender, age at diagnosis, tumor size, location, laboratory results, imaging findings, pathological details, treatment history, and outcomes, were systematically analyzed.

Patients were grouped based on their initial treatment approach: radical surgery or radical radiotherapy. Additionally, those who experienced recurrence were further divided into two subgroups: those who underwent salvage surgery and those who received salvage radiotherapy.

A total of 26 patients received 22 rounds of radiotherapy, utilizing either IMRT or VMAT technology. The gross tumor volume (GTV) included the visible tumor or postoperative bed, as seen in positioning CT or MR images. The clinical target volume (CTV) extended from the GTV, encompassing relevant lymphatic drainage areas, while the planning target volume (PTV) was expanded by 3–5 mm from the CTV. Organs at risk were also delineated. The median dose for 95% of the PTV was 36.0Gy, with a dose range of 28.0-40.0 Gy administered in 14–20 fractions (2.0-2.12Gy per fraction, 1 fraction per day, 5 days per week).

The patients were followed up until March 2024, with follow-up durations ranging from 17 to 168 months (median: 86 months). Follow-up was conducted through outpatient visits and telephone consultations, focusing on survival status and disease recurrence or progression.

Locoregional control was assessed based on clinical and radiographic evaluations. Tumors were considered controlled if there was at least a partial response (50% or greater decrease in the largest tumor diameter) with no evidence of regrowth. Local recurrence was defined as any increase in tumor size or recurrence within the surgical bed, radiation field, or at the irradiated area margins, as well as in the affected anatomical compartment.

### 2.1 Statistical analysis

Statistical analysis was performed using SPSS version 25.0 and R 4.1.1, with a significance threshold set at p < 0.05 (two-sided). Univariate analysis assessed the relationship between clinical and treatment-related variables and recurrence rates. Continuous variables were compared using t-tests, while categorical variables were analyzed with chi-square or Fisher’s exact tests, as appropriate. Kaplan-Meier (K-M)survival curves were generated for each factor, and differences between groups were compared using the log-rank test. For multivariate analysis, all variables identified as significant in the univariate analysis were included in a Cox proportional hazards regression model to identify independent risk factors for recurrence. Hazard ratios (HRs) with 95% confidence intervals (CIs) were calculated for each variable. The proportional hazards assumption was tested and satisfied.

## 3 Result

### 3.1 Basic information of all patients

A total of 26 patients were included in the study, of which 24 were male and 2 were female, with a mean age of 42.2 ± 17.7 years. Among them, 19 patients received primary surgery (PS), 7 patients received primary radiotherapy (PR). The tumor diameter ranged from 2 to 9.3 cm, with an average of 4.66 ± 1.77 cm. Multiple tumors were found in 18 cases (69.2%), and bilateral involvement occurred in 8 cases (30.8%). Most lesions were localized in the head and neck regions, with the parotid and submandibular areas being the most affected The detailed characteristics are shown in **Table 1**.

**Table 1:** Basic information of all patients.

ID	Group	Age	Gender	Initial location	Maximum diameter (cm)	Single/ Sultiple (1/2)	Unilateral/ Bilatera (1/2)
KD01	PS+S/R	59	Female	Parotid gland/LN	2	2	1
KD02	PS+S/R	34	Male	Parotid gland /Postauricular	2.5	2	2
KD03	PS+S/R	75	Male	Temporal /Eye socket	4.5	2	1
KD04	PS+S/R	21	Male	Cheek/LN	4	2	1
KD05	PS+S/R	39	Male	Parotid gland	4.4	1	1
KD06	PS+S/R	19	Male	Parotid gland /Postauricular	4.5	2	1
KD07	PS+S/R	19	Male	Parotid gland/LN	2.5	2	2
KD08	PS+S/R	51	Female	Postauricular/LN	5	2	1
KD09	PS+S/R	50	Male	Parotid gland	5.5	1	1
KD10	PS+S/R	62	Male	Parotid gland	8	1	1
KD11	PS+S/R	45	Male	Nasal cavity	4.5	1	1
KD12	PS+S/R	63	Male	Parotid gland/LN	6	2	2
KD13	PS+S/R	34	Male	Parotid gland	2.5	2	2
KD14	PS+S/R	41	Male	Parotid gland	6	1	1
KD15	PR	35	Male	Parotid gland/LN	7.5	2	1
KD16	PR	39	Male	Postauricular/LN	5	2	1
KD17	PR	68	Male	Cheek	4	2	1
KD18	PR	49	Male	Postauricular	4	2	2
KD19	PR	31	Male	Parotid gland/LN	5.6	2	2
KD20	PR	66	Male	Cheek	3.7	2	1
KD21	PR	40	Male	Parotid gland	4.4	1	1
KD22	PS	46	Male	Submandibular	9.3	1	1
KD23	PS	17	Male	Submandibular /Parotid gland	5	2	2
KD24	PS	55	Male	Submandibular	3.5	2	1
KD25	PS	37	Male	Parotid gland	5.5	1	1
KD26	PS	3	Male	Clavicle/LN	2	2	2

PS, Primary surgery; PR, Primary radiotherapy; S/R, Surgery or Radiotherapy; LN, Lymph node.

### 3.2 Local control rates: primary surgery versus primary radiotherapy

**Figure 1:**
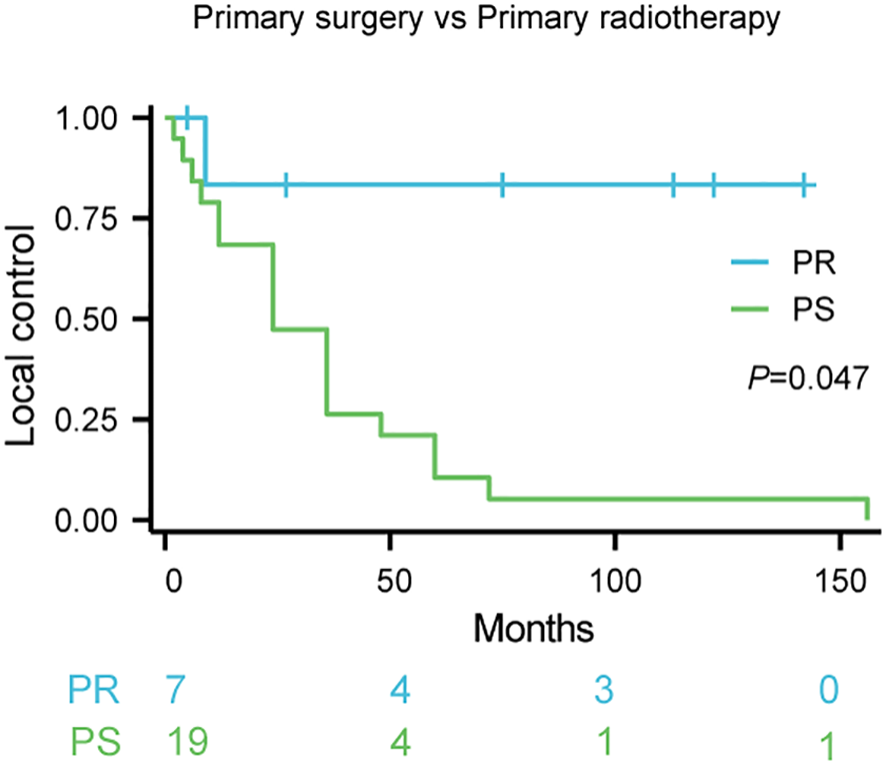
Local control rates following primary therapy for Kimura disease. Comparison of local control rates between primary surgery (PS) and primary radiotherapy (PR) in patients with Kimura disease. Kaplan-Meier survival curves demonstrate the proportion of patients achieving local control over time for each treatment group. The blue line represents PR (n=7), and the red line represents PS (n=19). The log-rank test revealed a statistically significant difference between the two groups (p=0.047). The numbers below the x-axis indicate the number of patients remaining at risk in each group at specific time points during the follow-up period.

K-M survival analysis revealed a statistically significant difference in local control rates between patients treated with primary surgery (PS) and those receiving primary radiotherapy (PR) (p = 0.047; **Figure 1**). The estimated 5-year local control rate for PR was 75%, compared to 15% for PS. The survival curves demonstrate that primary radiotherapy resulted in superior local control compared to primary surgery, suggesting that radiotherapy may be a more effective initial treatment strategy for achieving optimal local disease management in Kimura disease.

### 3.3 Local control rates: salvage surgery versus salvage radiotherapy

**Figure 2:**
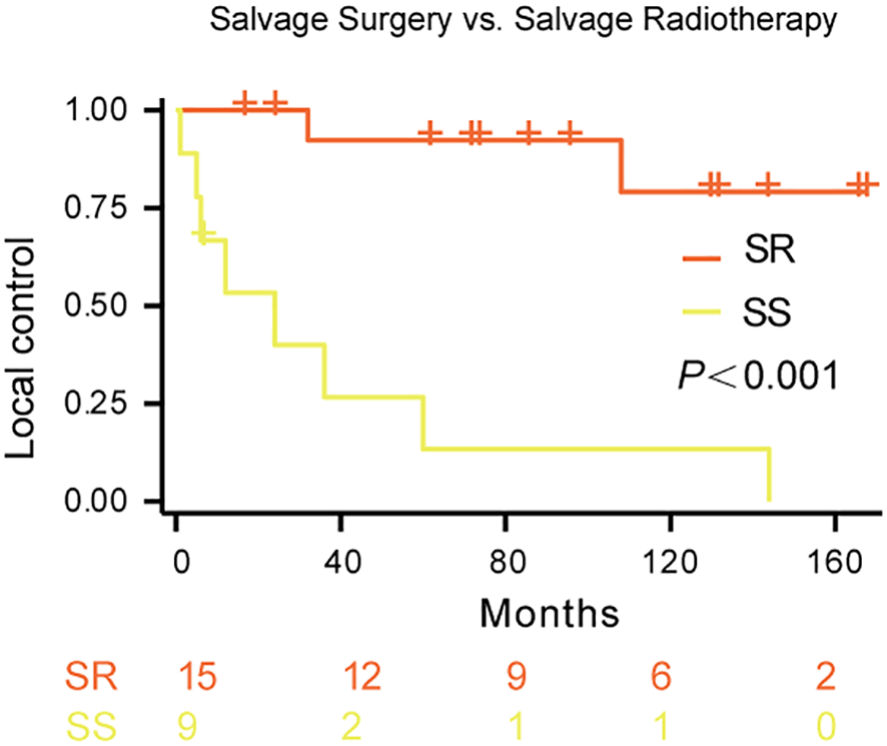
Local control rates following salvage therapy for recurrent Kimura disease. Comparison of local control rates between salvage radiotherapy (SR) and salvage surgery (SS) in patients with recurrent Kimura disease. Kaplan-Meier survival curves demonstrate the proportion of patients achieving local control over time for each treatment group. The blue line represents SR (n=15), and the red line represents SS (n=9). The log-rank test revealed a statistically significant difference between the two groups (p<0.001).

K-Meier survival analysis demonstrated a significantly higher local control rate with salvage radiotherapy (SR) compared to salvage surgery (SS) in patients with recurrent Kimura disease (p < 0.001; **Figure 2**). The estimated 5-year local control rate for SR was approximately 80%, while the 5-year local control rate for SS was approximately 0%. The survival curves strongly suggest that salvage radiotherapy is superior to salvage surgery for achieving local control in recurrent Kimura disease.

### 3.4 Local control rates: primary radiotherapy versus salvage radiotherapy

**Figure 3:**
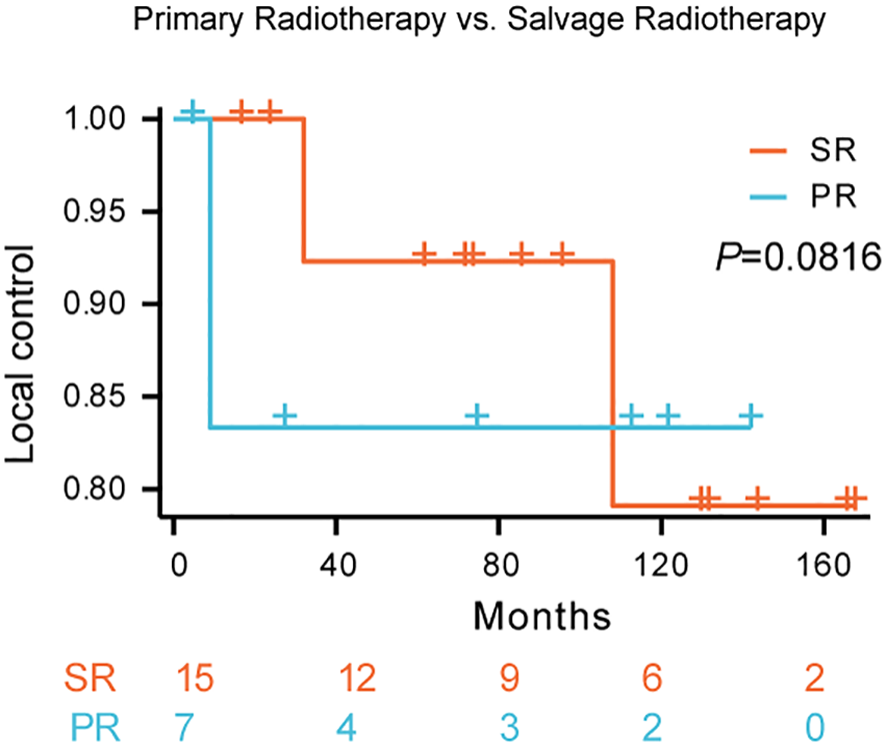
Local control rates following primary radiotherapy versus salvage radiotherapy for Kimura disease. Comparison of local control rates between primary radiotherapy (PR) and salvage radiotherapy (SR) in patients with Kimura disease. Kaplan-Meier survival curves demonstrate the proportion of patients achieving local control over time for each treatment group. The blue line represents SR, and the red line represents PR. The log-rank test did not reveal a statistically significant difference between the two groups (p=0.0816).

K-M survival analysis did not reveal a statistically significant difference in local control rates between patients treated with primary radiotherapy (PR) and those receiving salvage radiotherapy (SR) (p = 0.0816; **Figure 3**). The estimated 5-year local control rate for SR was approximately 79%, compared to approximately 83% for PR. While the survival curves suggest a trend towards improved local control with primary radiotherapy, this difference did not reach statistical significance.

### 3.5 Univariate analysis of factors affecting local control rate

**Figure 4:**
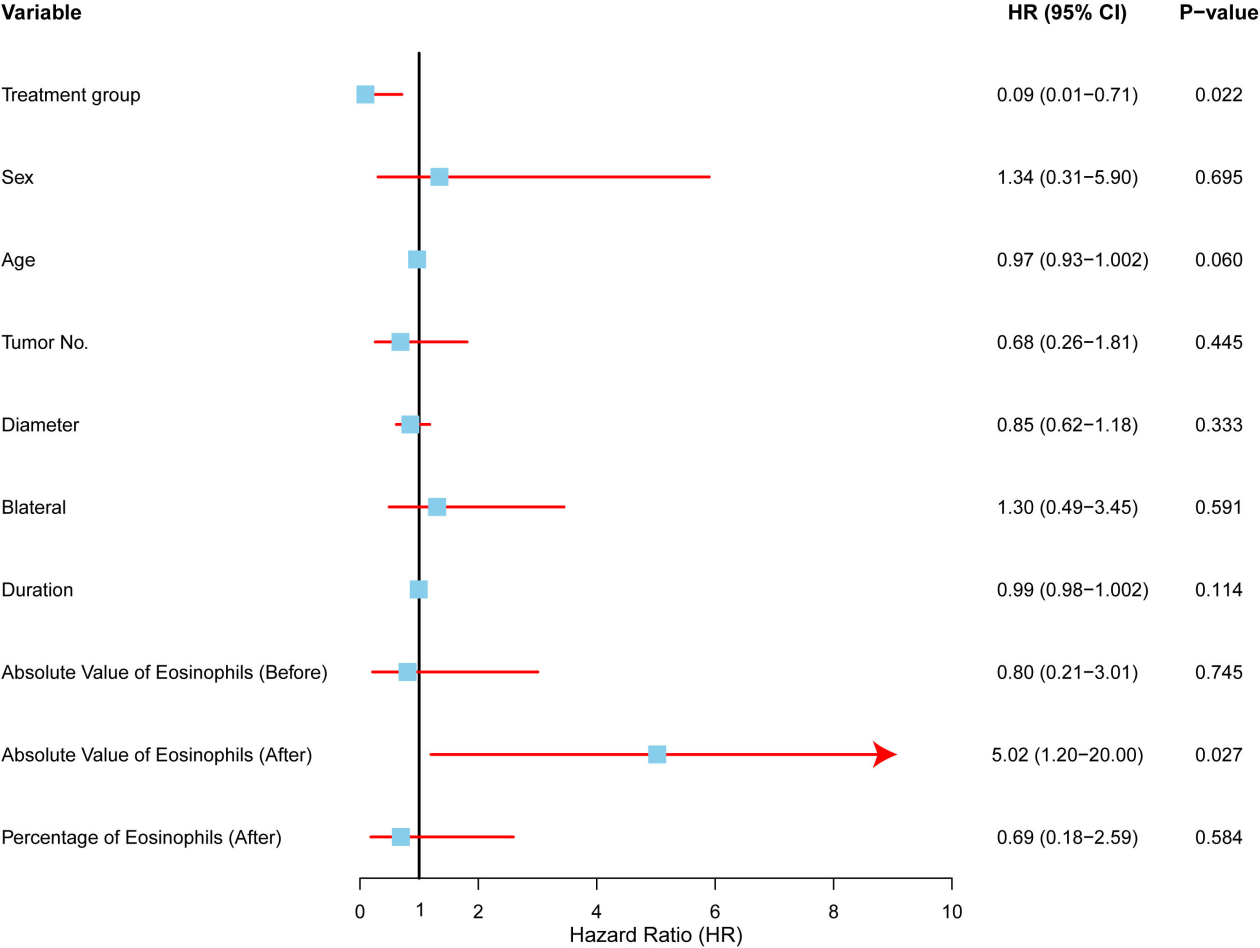
Univariate analysis of factors affecting local control in Kimura disease. Forest plot depicting hazard ratios (HRs) and 95% confidence intervals (CIs) for each variable included in the univariate Cox regression analysis. The vertical line represents HR = 1.0. Variables with HRs significantly different from 1.0 (p < 0.05) are considered to be associated with local control. RT (radiotherapy) was associated with improved local control compared to SURG (surgery), while elevated post-treatment absolute eosinophil counts were associated with decreased local control.

Univariate Cox regression analysis was performed to identify factors associated with local control in Kimura disease (**Figure 4**). The forest plot summarizes the hazard ratios (HRs) and 95% confidence intervals (CIs) for each variable. The analysis revealed that treatment group and post-treatment eosinophil count were significantly associated with local control. Patients receiving radiotherapy (RT) demonstrated significantly improved local control compared to those undergoing surgery (SURG), with a hazard ratio (HR) of 0.093 (95% CI: 0.012–0.709, p=0.022). Conversely, elevated post-treatment absolute eosinophil counts were associated with a significantly decreased local control rate (HR = 5.020, 95% CI: 1.200–20.994, p=0.027). Other factors, including sex, age, tumor number, tumor diameter, bilaterality, and symptom duration, did not show statistically significant associations with local control in this univariate analysis.

### 3.6 Multivariate analysis of factors affecting local control rate

**Figure 5:**
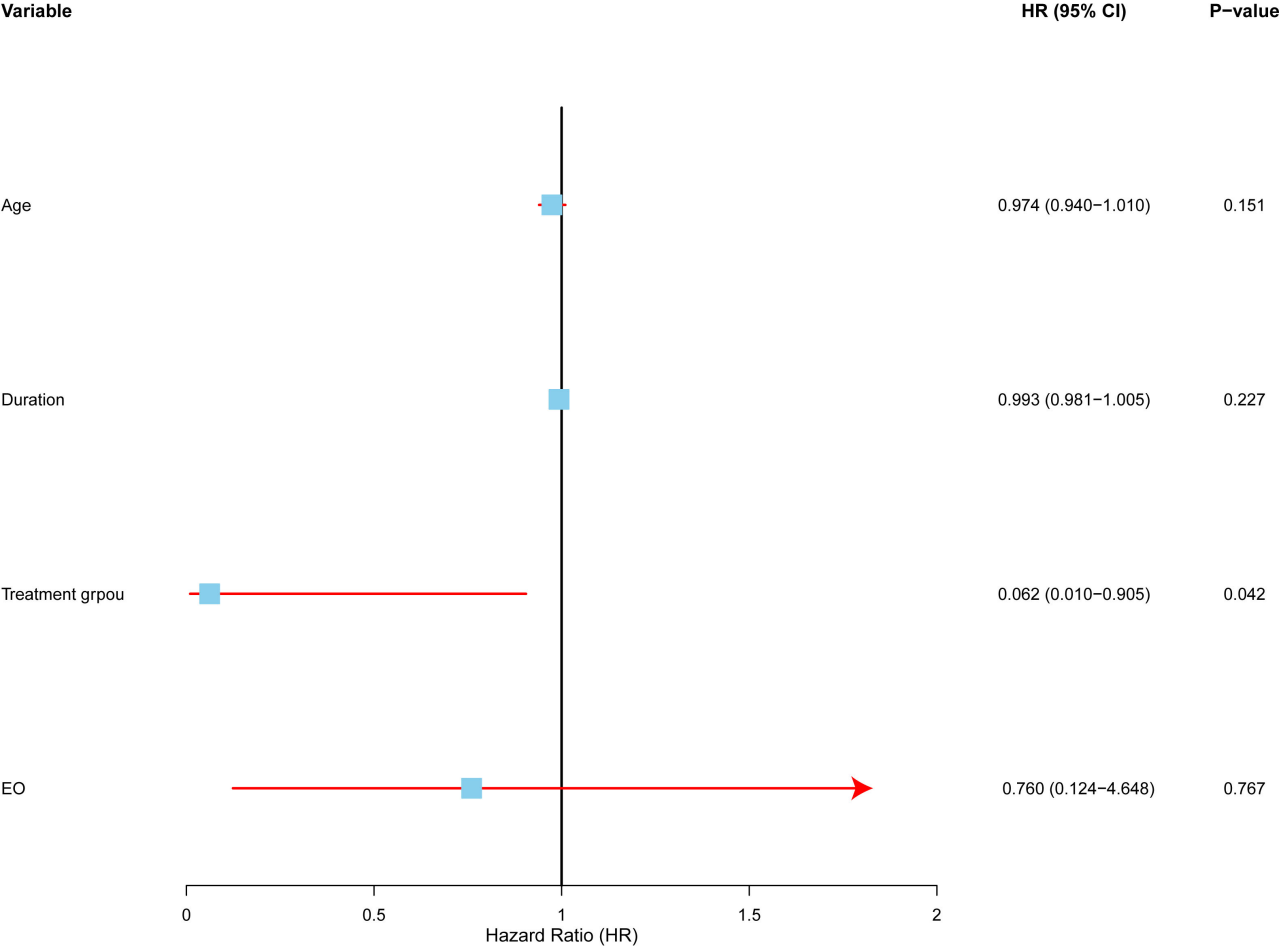
Multivariate analysis of factors affecting local control in Kimura disease. Forest plot depicting hazard ratios (HRs) and 95% confidence intervals (CIs) for each variable included in the multivariate Cox proportional hazards regression model. The model adjusted for age, symptom duration, and post-treatment eosinophil count. The vertical line represents HR = 1.0. Treatment modality (RT vs. SURG) was the only independent predictor of local control, with radiotherapy demonstrating a significantly lower hazard ratio.

To identify independent predictors of local control, we conducted a multivariate Cox proportional hazards analysis (**Figure 5**).The analysis included four variables: treatment modality (surgery vs. radiotherapy), age, symptom duration, and post-treatment eosinophil count (low vs. high).

The results revealed that treatment modality was the only statistically significant independent risk factor for local control. Radiotherapy (RT) was associated with a higher local control rate than surgery (SURG), with a hazard ratio (HR) of 0.062 (95% CI: 0.004–0.905, p=0.042). This suggests that radiotherapy reduces the risk of local recurrence more effectively than surgery.

### 3.7 Data expansion and categorization

To address the limited number of treated patients, we expanded the dataset by considering each treatment episode as a separate observation.For example, if a patient underwent surgery, it was recorded as one treatment event. If the same patient experienced a recurrence and later received radiotherapy, it was recorded as a second event. For each treatment episode, we tracked the recurrence-free period following the specific treatment.Using this approach, we grouped all treatments into two categories: surgery and radiotherapy. Recurrence times were then analyzed separately for each group. This method increased the sample size for statistical analysis and provided a clearer understanding of recurrence patterns based on treatment type.To identify predictors of post-treatment recurrence, we included only pre-treatment and unrelated indicators in the subsequent analysis. Details of the included patient characteristics are provided in Appendix Table 1.

### 3.8 Multivariate analysis after data expansion: affecting local control in all patients

**Figure 6:**
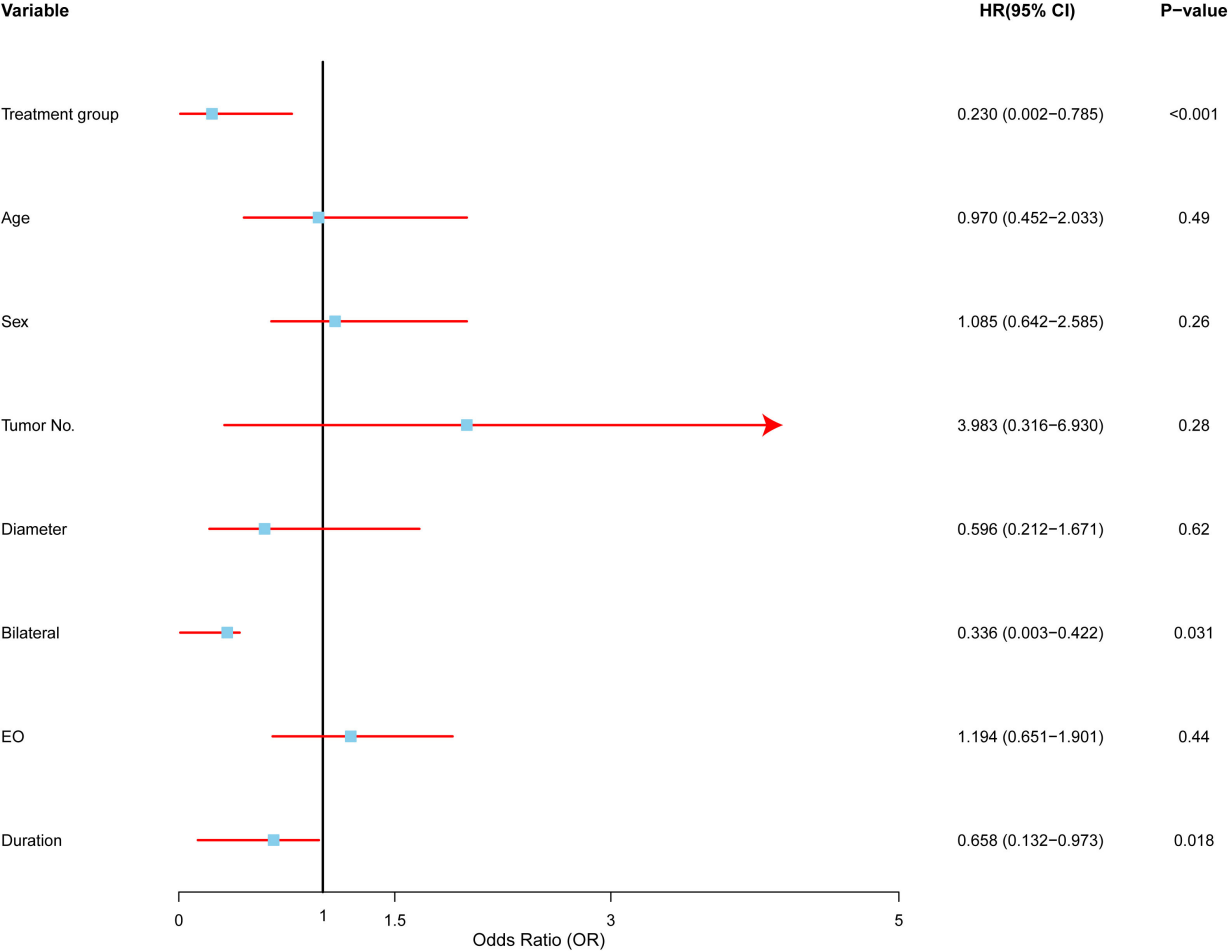
Multivariate analysis of factors affecting recurrence in Kimura disease. Forest plot depicting hazard ratios (HRs) and 95% confidence intervals (CIs) for each variable included in the multivariate Cox proportional hazards regression model. The vertical line represents HR = 1.0. Variables with HRs significantly different from 1.0 (p < 0.05) are considered to be independent predictors of recurrence. RT (radiotherapy) was associated with a lower risk of recurrence compared to SURG (surgery), multiple tumors were associated with a higher risk of recurrence, bilateral involvement was associated with a lower risk of recurrence, and longer symptom duration was associated with a lower risk of recurrence.

After expanding the dataset, 19 surgery and 22 radiotherapy patients were included in the study. We performed a multivariate Cox proportional hazards analysis (**Figure 6**) to identify key factors predicting recurrence post-treatment.

Treatment modality emerged as a significant predictor. Radiotherapy was associated with a substantially lower risk of recurrence compared to surgery (HR: 0.23, 95% CI: 0.0002–0.7850, p < 0.01), suggesting that radiotherapy offers better long-term control and may be preferred, particularly in high-risk cases.

The number of tumors was another important predictor. Patients with multiple tumors faced nearly four times the risk of recurrence compared to those with a single tumor (HR: 3.98, 95% CI: 0.3164–6.9300, p < 0.05), indicating that multiple tumors increase the likelihood of disease recurrence.

Bilateral involvement, or disease affecting both sides of the body, was linked to a lower risk of recurrence (HR: 0.336, 95% CI: 0.0027–0.4222, p < 0.05). This suggests that unilateral disease may represent a more aggressive form of Kimura disease, which may require more intensive treatment.

Interestingly, a longer duration of symptoms before treatment was associated with a lower risk of recurrence (HR: 0.6585, 95% CI: 0.1320–0.9725, p < 0.01), possibly indicating that a longer symptom duration correlates with a less aggressive disease course.

In contrast, factors such as age (HR: 0.97, p > 0.05), sex (HR: 1.08, p > 0.05), and tumor size (HR: 0.59, p > 0.05) did not show significant associations with recurrence, suggesting these may not be as reliable for predicting outcomes in Kimura disease.

### 3.9 Multivariate analysis after data expansion: affecting local control in radiotherapy patients

**Figure 7:**
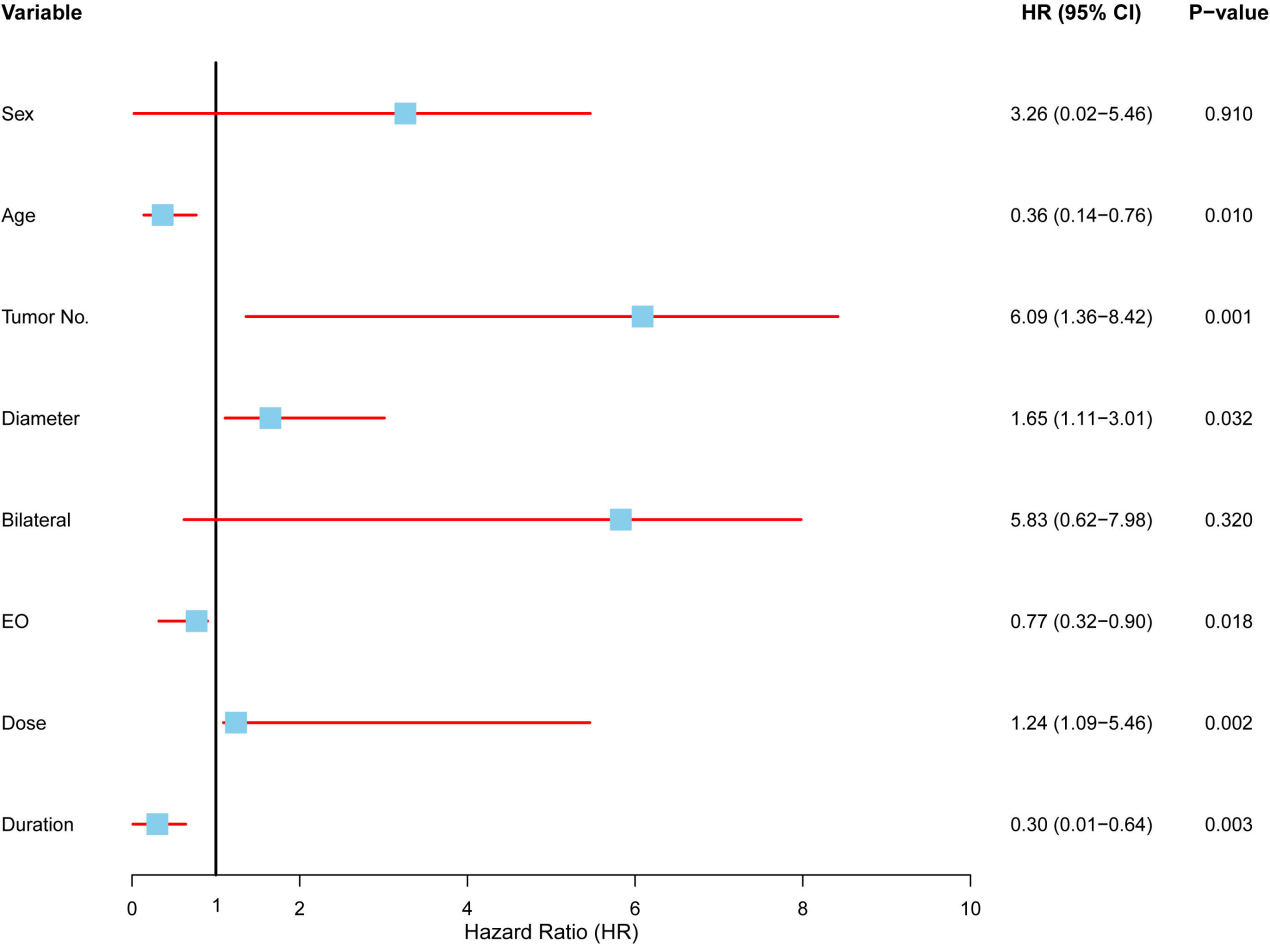
Multivariate analysis of factors affecting local control in radiotherapy patients. Forest plot displaying the results of a Cox proportional hazards model evaluating the association between clinical variables and local control in patients undergoing radiotherapy. Each variable is represented by a hazard ratio (HR) with corresponding 95% confidence intervals (CI) and p-values. Significant predictors included age (HR: 0.36, 95% CI: 0.14–0.76, p = 0.010), tumor number (HR: 6.09, 95% CI: 1.36–8.42, p = 0.001), tumor diameter (HR: 1.65, 95% CI: 1.11–3.01, p = 0.032), symptom duration (HR: 0.30, 95% CI: 0.01–0.64, p = 0.003), eosinophil count (HR: 0.77, 95% CI: 0.32–0.90, p = 0.018), and radiotherapy dose (HR: 1.24, 95% CI: 1.09–5.46, p = 0.002).

In our multivariate analysis group, several key factors emerged as strong predictors of recurrence, while others showed no significant association (**Figure 7**). The major findings include:

Age had a protective effect, with older patients exhibiting a lower risk of recurrence (HR = 0.36, 95% CI: 0.14–0.76, p = 0.01), suggesting that older patients are less likely to experience recurrence compared to younger ones. Tumor number was a crucial predictor, as patients with multiple tumors had a significantly higher risk of recurrence (HR = 6.09, 95% CI: 1.36–8.42, p = 0.001), highlighting the increased recurrence likelihood in those with multiple tumors undergoing radiotherapy. Tumor size also played a role, with larger tumors correlating to a higher recurrence risk (HR = 1.65, 95% CI: 1.11–3.01, p = 0.032). Interestingly, a longer duration of symptoms before treatment was associated with a reduced recurrence risk (HR = 0.30, 95% CI: 0.01–0.64, p = 0.003), suggesting that slower disease progression might result in better outcomes. Eosinophil counts and radiotherapy dose were also influential. Higher eosinophil levels prior to treatment were linked to better recurrence control (HR = 0.77, 95% CI: 0.32–0.90, p = 0.018), while lower radiotherapy doses were associated with improved outcomes (HR = 1.24, 95% CI: 1.09–5.46, p = 0.002), suggesting that higher radiotherapy intensities may not enhance recurrence control. In contrast, sex (HR = 3.26, p = 0.910) and bilateral disease (HR = 5.83, p = 0.320) did not show significant associations with recurrence risk. These factors displayed wide confidence intervals, indicating limited relevance for predicting recurrence in the radiotherapy group.

## 4 Discussion

This study highlights the crucial role of radiotherapy (RT) in managing Kimura disease (KD), demonstrating superior local control rates compared to both primary and salvage surgery. Our findings suggest that RT, whether as an initial treatment or after recurrence, significantly reduces disease relapse, with local control rates exceeding 80% in both cases. Multivariate analysis identified RT as the most influential factor in preventing recurrence, reinforcing its efficacy for long-term disease control. These results align with previous studies, such as Chang et al. (14), which reported local control was achieved in 64.3% (9/14) of the RT group compared to 22.2% (2/9) of the non-RT group. The advantage of RT likely stems from its ability to eradicate residual microscopic disease that may evade surgical removal, offering a more comprehensive disease management strategy.

The superior efficacy of radiotherapy over surgery may be explained by several biological mechanisms. While surgery aims to remove macroscopic disease, it may not address the underlying immunological dysregulation that characterizes KD. Radiotherapy appears to exert its beneficial effects through modulation of the inflammatory microenvironment by: (1) reducing the proliferation and activation of T-helper cells that drive the eosinophilic inflammation (15); (2) decreasing the production of cytokines such as IL-4, IL-5, and IL-13 that promote eosinophil recruitment and activation (12), Additionally, moderate-dose radiotherapy may have immunomodulatory effects that extend beyond the direct radiation field, potentially addressing microscopic disease extensions that surgery might miss.

Interestingly, our study found that moderate RT doses (30.0–40.0 Gy) were as effective as higher doses, challenging the assumption that dose escalation improves outcomes. This finding is supported by Hareyama et al. (16)and Fionda et al. (17), who observed optimal local control with 26.0–30.0 Gy and no added benefit from higher doses. Clinically, this suggests that moderate doses can achieve effective disease control while minimizing radiation-induced toxicity, such as fibrosis and other long-term complications.

An unexpected finding was the association between longer symptom duration before treatment and lower recurrence rates in RT patients. This contrasts with Lee et al. (18), who reported better surgical outcomes in patients with shorter symptom durations (<5 years). Our data suggest that RT may be more effective in chronic or stable disease, potentially due to its ability to target well-defined lesions within a chronic inflammatory environment. However, Surgery’s effectiveness likely diminishes over time as lesions grow more extensive and infiltrative, increasing the difficulty of achieving complete excision. These findings underscore the differing mechanisms of RT and surgery, emphasizing the need for individualized treatment strategies based on disease progression.

Tumor number and size also emerged as significant predictors of recurrence in RT patients. Larger tumors (HR: 1.65) and multiple lesions (HR: 6.09) correlated with poorer outcomes, consistent with Chen et al. (19), who found similar trends in surgical cases. While Chen suggested that tumor burden may not directly impact recurrence if surgery is successful, our study indicates that in RT, larger or multiple tumors pose challenges for effective treatment planning and radiation delivery. This reinforces the importance of tailoring RT protocols to tumor characteristics.

Additionally, we identified eosinophil (EO) counts as a predictor of recurrence. Lower post-treatment EO levels correlated with reduced recurrence, supporting findings by Liu et al. (20) and Kim et al. (21) on the role of eosinophils in KD pathophysiology. However, in the RT subgroup, higher pre-treatment EO levels were associated with better local control. This suggests that eosinophilic infiltrates may be more responsive to radiation or that elevated EO levels indicate a stronger immune response, enhancing RT efficacy. These findings highlight the complex role of eosinophils in KD and warrant further investigation into their prognostic value.

Despite its strengths, this study has limitations. Its retrospective nature introduces potential selection bias, particularly in treatment assignments. Additionally, We acknowledge the inherent limitations of our study’s small sample size (26 patients), which reflects the rarity of Kimura disease. Our approach of treating each recurrence as an independent event was implemented to increase statistical power for analysis; however, we recognize this may introduce statistical bias. Nevertheless, this methodological approach represents a compromise necessitated by the rarity of the disease, and our findings should be interpreted with appropriate caution. Furthermore, variability in RT doses and techniques across patients could have influenced outcomes. Future prospective, multicenter trials with standardized RT protocols are needed to validate our findings.

Future research should focus on randomized controlled trials to confirm the superiority of RT over surgery, particularly in recurrent or multifocal KD cases. Longer follow-up periods are necessary to assess RT’s long-term efficacy and safety, including potential late recurrences or radiation-induced complications. Optimizing RT dose protocols should also be a priority, balancing efficacy with toxicity reduction.

Additionally, further studies should explore the biological mechanisms underlying the observed correlations between eosinophil counts and treatment outcomes. Immunohistochemical analyses of eosinophils, IgE, and other immune markers could help identify predictive biomarkers for RT response and prognosis. Molecular profiling of tumors may also uncover genetic or immune pathways influencing RT efficacy, potentially guiding personalized treatment approaches.

## 5 Conclusion

This study reinforces RT as a highly effective treatment for KD, particularly in recurrent or multifocal cases. Our findings highlight the need for personalized treatment strategies that consider tumor characteristics and disease duration. Future research should aim to optimize RT protocols and further explore the immune mechanisms influencing KD progression and treatment response.

## Data Availability

The raw data supporting the conclusions of this article will be made available by the authors, without undue reservation.
